# Pharmacological Effects of Shikonin and Its Potential in Skin Repair: A Review

**DOI:** 10.3390/molecules28247950

**Published:** 2023-12-05

**Authors:** Yanping Song, Qiteng Ding, Yuewen Hao, Bing Cui, Chuanbo Ding, Feng Gao

**Affiliations:** 1 College of Traditional Chinese Medicine, Jilin Agriculture Science and Technology University, Jilin 132101, China; 15083339965@163.com; 2 College of Chinese Medicinal Materials, Jilin Agricultural University, Changchun 130118, China; ding152778@163.com; 3 Jilin Jianwei Natural Biotechnology Co., Ltd., Linjiang 134600, China; w2023@163.com (Y.H.); cb1369@126.com (B.C.); 4 Jilin Aodong Yanbian Pharmaceutical Co., Ltd., Dunhua 133700, China

**Keywords:** Shikonin, dermatophytosis, biological activity

## Abstract

Currently, skin injuries have a serious impact on people’s lives and socio-economic stress. Shikonin, a naphthoquinone compound derived from the root of the traditional Chinese medicine Shikonin, has favorable biological activities such as anti-inflammatory, antibacterial, immunomodulatory, anticancer, and wound-healing-promoting pharmacological activities. It has been reported that Shikonin can be used for repairing skin diseases due to its wide range of pharmacological effects. Moreover, the antimicrobial activity of Shikonin can play a great role in food and can also reduce the number of pathogenic bacteria in food. This paper summarizes the research on the pharmacological effects of Shikonin in recent years, as well as research on the mechanism of action of Shikonin in the treatment of certain skin diseases, to provide certain theoretical references for the clinical application of Shikonin, and also to provides research ideas for the investigation of the mechanism of action of Shikonin in other skin diseases.

## 1. Introduction

**Figure 1:**
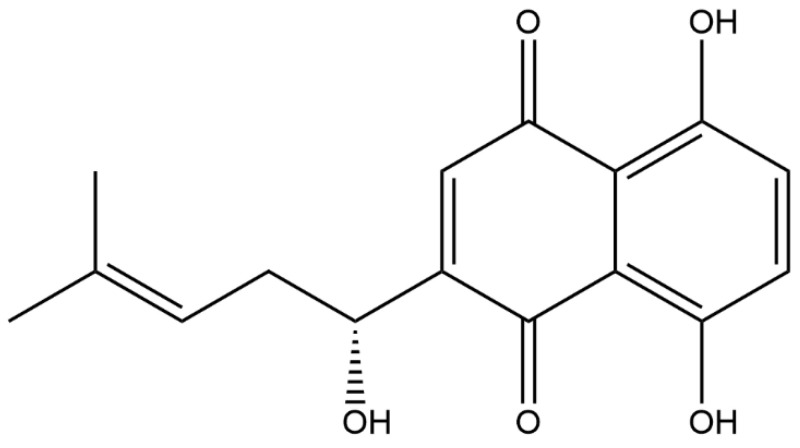
Chemical structural formula of Shikonin.

*Lithospermum erythrorhizon*, a traditional Chinese medicine, is known for its sweet, salty, and cooling taste. It primarily regulates the heart and liver meridians, offering various benefits such as bringing down heat, cooling the blood, detoxifying, and minimizing rashes. As an active ingredient in *Lithospermum erythrorhizon*, the molecular formula of Shikonin is C_16_H_16_O_5_ (Figure 1), which is a kind of natural naphthoquinone extracted from the root of *Lithospermum erythrorhizon*. *Lithospermum erythrorhizon*, also known as lithospermum paste, was first recorded in the classic Chinese medical book *Shennong’s Herbal Classic*. Since ancient times, it has been widely used to reduce body surface heat, promote blood circulation, clear congestion, and detoxify. In addition, other diseases, such as macula, measles, sore throat and carbuncle, have also been treated using comfrey paste. In recent decades, it has not only been applied in different Chinese herbal formulations to treat various internal diseases but also made into topical drugs for the treatment of skin diseases [1]. Shikonin has many pharmacological properties, including anti-inflammatory, antioxidant, antibacterial, and anti-tumor properties, ameliorating of skin diseases and having other pharmacological effects [2,3,4,5,6,7,8].

Shikonin is a red naphthoquinone compound that is abundant in the roots of the Shikonaceae plant. It is not a specific compound but rather refers to a group of compounds. The parent nucleus of Shikonin is 5,8-dihydroxy-1,4-naphthoquinone, and it has a hydroxy-substituted isohexenyl side chain. Shikonin compounds are divided into two optical isomers, L-Shikonin (alkannin, S type) and D-Shikonin (Shikonin, R type), based on their different optical activities. Shikonin exists in balance with its enantiomer alkannin; hence, it is called A/S, and has shown various pharmacological activities [9]. For Shikonin, the commonly used extraction methods include solvent extraction, ultrasonic extraction, etc. Huang et al. [10] used the response surface method (RSM) to optimize the method of ultrasonic-assisted extraction (UAE) to extract Shikonin from *Arnebia euchroma*, and found the best extraction conditions for Shikonin: the ultrasonic power was 93 W, the time was 87 min, the temperature was 39 °C, and the liquid–solid ratio was 11:1. Homogenate extraction is a convenient, rapid, and efficient sample preparation technology that can be used for the extraction of Shikoin. Liu et al. [11] conducted a three-factor, three-level experimental design using the Box–Behnken design method, based on single-factor experiments. The optimal extraction conditions were determined to be using 78% ethanol as a solvent, an extraction time of 4.2 min, a liquid–solid ratio of 10.3, and two extraction cycles. Microwave-assisted extraction of Shikonin was found to be a rapid and effective method. A central composite design approach was used to optimize several variables that could affect the efficiency of microwave-assisted extraction, including temperature, the methanol concentration in the extraction solvent mixture, extraction time, and solvent volume. The results showed that temperature and the methanol concentration in the extraction solvent mixture were the most important factors [12].

The skin is a complex organ composed of three layers: the epidermis, dermis, and subcutaneous tissue. As the largest organ in the human body, it serves as both a protective barrier and a stress response organ. The skin also plays a crucial role in maintaining the stability of the body’s internal environment. These functions are regulated by various signal molecules produced by the local neuroendocrine and immune systems, as well as resident and immune cells [13]. When its skin is damaged, an organism will experience a series of complex, coordinated processes involving various cellular components, including inflammatory cells and tissue repair cells, to promote the healing of skin trauma. Shikonin has a wide range of pharmacological activities that can treat related skin diseases. In recent years, there has been significant research on the effects of Shikonin in repairing skin damage and treating various skin diseases, including hepatocellular carcinoma, scarring, psoriasis, and dermatitis. The extensive biological activities of Shikonin and its application in skin diseases have garnered widespread attention. Therefore, this paper summarizes the extensive pharmacological activities of Shikonin and the related research progress in skin diseases to provide theoretical references for subsequent applications.

## 2. Biological Activity

The pharmacological effects of Shikonin have been studied quite extensively in recent years, which provides a prerequisite for the better development of Shikonin.

### 2.1. Anti-Inflammatory

Inflammatory reactions in the body can lead to the occurrence of various diseases. A large body of research now shows that Shikonin has significant anti-inflammatory effects. For example, Shikonin reduces the incidence of arthritis and relieves joint inflammation. It exerts anti-inflammatory effects by inhibiting the conversion of M1 macrophages into M2 macrophages in the joint tissues of mice with collagen-induced arthritis (CIA). It inhibits the inflammatory response in the joint tissues of mice [14]. In an interleukin (IL)-1β-induced cellular model of osteoarthritis, purslane treatment reduced the mRNA and protein levels of thrombospondin motif-5, the disintegrin of the matrix metalloproteinase-1, and metalloproteinases. In addition, after analysis using the Osteoarthritis Research Society International (OARSI) score, it was found that the symptoms of cartilage degeneration were reduced after Shikonin treatment in mice [15]. In sepsis, Shikonin improved sepsis-induced lung injury by modulating the miRNA-140-5p/toll-like receptor-4 (TLR4) pathway [16]. Shikonin also inhibits microglial cell morphology changes, thereby ameliorating the pain response induced by mechanical stimuli [17]. In ulcerative colitis, Shikonin enhances the cell migration of intestinal epithelial cells via a mechanism involving transforming growth factor-β1 (TGF-β1) induction for the treatment of colitis [18]. In a previous study, it was shown that self-assembled nanogels containing comfreyin have potent anti-inflammatory effects that can be mediated by modulating the inflammatory response of the innate immune cells and the activation of the NOD-like receptor protein 3 (NRLP3) inflammatory vesicles [19]. The anti-inflammatory mechanisms of Shikonin are varied and can be used to treat not only arthritis, which manifests on the outside of the skin, but also sepsis, which manifests on the inside of the skin. With the wide range of anti-inflammatory effects and diverse anti-inflammatory mechanisms, the study of the anti-inflammatory effects of Shikonin is the key to our treatment of other conditions.

### 2.2. Antimicrobial

Bacteria are types of microorganisms that are commonly found in nature and are ubiquitous. Certain pathogenic bacteria can infect the human body, leading to various diseases. For instance, *Glucococcus* can cause septic diseases, *Diplococcus* can cause gonorrhea and meningitis, and *Salmonella* can result in typhoid fever. Other bacterial infections can lead to diseases such as pneumonia, tuberculosis, peritonitis, enteritis, and gastritis. Shikonin can be combined with a variety of polymer materials to exert antibacterial effects. Electrostatic spinning is a versatile method for producing ultrathin fibers with desirable properties, and the technique can be optimized by controlling parameters such as the solution/melt viscosity, feed rate, and electric field voltage. Maliszewska et al. [20] used electrostatic spinning to prepare composites containing Shikonin with antimicrobial properties.

Polymer fibers can be effective antimicrobials. Hydroxypropyltrimethyl ammonium chloride chitosan/polycaprolactone/Shikonin nanofiber membranes have better hydrophobicity, barrier properties, and mechanical properties. An HACC/PCL membrane containing 2 wt% Shikonin (SK) (alizarin) showed a good antimicrobial effect within 24 h [21]. Arampatzis et al. [22] developed electrospun scaffolds that could be used as carriers of bioactive natural products alkanin and alizarin (A/S), and successfully prepared a series of polymer nanofibers consisting of cellulose acetate (CA) or poly(ε-caprolactone) (PCL) and different ratios of the A/S derivative mixture. Polymer nanofibers, in an electrospun scaffold, significantly inhibited the growth of *Staphylococcus epidermidis* and *Staphylococcus aureus* around the edges of the fiber mats. Priyadarshi et al. [23] first reviewed the progress of Shikonin as a multifunctional material in biopolymer films for smart packaging, which in turn reviewed the incorporation of Shikonin into the films as an active substance with good antibacterial properties. It has been found that the effect of Shikonin on anti-methicillin-resistant Staphylococcus aureus is related to its affinity for peptidoglycan, the permeability of the cytoplasmic membrane, and the ATP-binding cassette (ABC) transporter activity [24].

**Figure 2:**
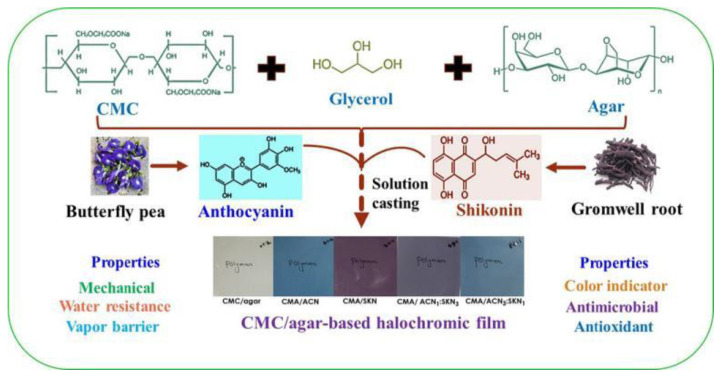
Schematic diagram of the fabrication of carboxymethyl cellulose (CMC)/agar-based functional color-changing membranes [26].

The antimicrobial effect of Shikonin also has great potential in food packaging. A study has prepared a multifunctional carboxymethyl cellulose/agar smart membrane by combining cellulose nanocrystals (CNC) separated from onion skin with Shikonin isolated from Shikonin roots. The composite membrane has strong antibacterial and mechanical properties and has great potential in practical food packaging applications [25]. Another smart membrane such as carboxymethyl cellulose (CMC)/agar-based functional color-changing membrane (Figure 2) exhibited significant pH-responsive color-changing performance in a pH range of 2–12, showing excellent acid-base gas-sensitive performance. With the addition of viologen, the membrane exhibited strong bacteriostatic activity and antioxidant activity against foodborne pathogenic bacteria. This color-developing film also has good mechanical properties and antibacterial functions, and has the potential for use in active and intelligent food packaging [26]. Gelatin/carrageenan-based functional smart films with good gas sensitivity and color stability were prepared from Shikonin and propolis. The combination of propolis and Shikonin led to the development of gelatin/carrageenan composite films that exhibited outstanding antimicrobial and antioxidant properties, and these smart films were effectively used for monitoring the freshness of packaged milk [27].

In food, foodborne *Staphylococcus aureus* (*S. aureus*) has attracted widespread attention for its role in foodborne infections and food poisoning in humans. In the range of 35–70 μg/mL, the minimum inhibitory concentrations (mic) of Shikonin were equal to the minimum bactericidal concentrations (MBCs). Shikonin inhibited the growth of *S. aureus* by decreasing the intracellular ATP concentration, hyperpolarizing the cell membrane, disrupting the integrity of the cell membrane, and altering the cell morphology. At non-inhibitory concentrations (NICs), Shikonin significantly inhibited *S. aureus* biofilm formation, which may be related to inhibiting the expression of the cidA and sarA genes. In addition, Shikonin significantly inhibited the transcription and expression of *S. aureus* virulence genes (sea and HLA) [28].

**Figure 3:**
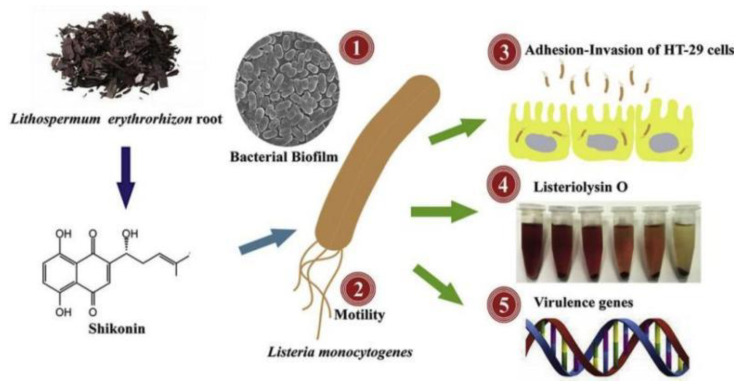
Transcriptional repression of related genes by Shikonin [30].

Shikonin showed potent in vitro antifungal activity against other pathogenic fungi such as *Candida*, *Aspergillus*, *Cryptococcus*, and *S. dermatophilus*, but showed no significant toxicity to mammalian cells, suggesting that SK is safe as a potential antifungal agent. Comedones also induced a range of apoptotic features, including phosphatidylserine externalization, chromatin condensation and fragmentation, reduced cytochrome c oxidase activity, and cysteoaspartase activation [29]. The lowest inhibitory concentration of Shikonin against *Listeria monocytogenes* was 25–100 μg/mL. Shikonin effectively reduced the ability of Lactobacillus monocytogenes to adhere to and invade HT-29 cells. In addition, Shikonin repressed the transcription of biofilm-related genes and infection-critical virulence genes (Figure 3) [30].

Gentamicin causes renal injury by accumulating in the proximal tubular epithelial cells via the megalin/cubilin/CLC-5 complex. Shikonin has been shown to have potential anti-inflammatory, antioxidant, antibacterial, and chloride channel inhibitory effects. Shikonin significantly and dose-dependently attenuated gentamicin-induced renal injury by restoring normal renal function and organization. Shikonin also restored intrarenal phagocytosis as evidenced by the inhibition of the elevation of renal macrophages, cubilin, and chloride channel 5 (CLC-5), and enhanced the gentamicin-induced reduction in sodium-hydrogen exchanger 3 (NHE3) levels and mRNA expression. Thus, Shikonin is a promising drug for the treatment of gentamicin kidney injury. Extensive research results have shown that Shikonin has a favorable inhibitory effect on a wide range of fungi and bacteria, which could play an important role not only in skin injury but also has great potential in food packaging and improving food safety [31].

### 2.3. Antiviral

Viruses, which are generally formed via the processes of adsorption, injection, multiplication, assembly, and release, are non-cellular microorganisms consisting of a long chain of nucleic acids and a protein shell. Once a virus enters a host cell, it can utilize the material and energy in the cell, as well as its ability to replicate, transcribe, and translate, to produce a new generation of viruses based on the genetic information contained in its nucleic acid. Antiviral mechanisms include inhibition of the host cell surface proteins, inhibition of their transcriptional and replicative abilities, and inhibition of apoptosis in infected cells [32]. Studies have shown that Shikonin can inhibit human immunodeficiency virus (HIV) invasion by 97–100% at non-cytotoxic levels in vitro. Chen et al. [33] discovered that Shikonin possesses the capability to interfere with downstream signaling, thereby obstructing chemokine receptors and inhibiting the replication ability of HIV-1. The investigation into the antiviral effects of Shikonin offers novel insights for the study of potential antiviral agents, particularly in the context of HIV treatment. Gong et al. [34] and Mao et al. [35] found that Shikonin could activate reactive oxygen species and c-Jun amino-terminal kinase to induce cell apoptosis. Moon et al. [36] further found that Shikonin can induce ER stress by producing reactive oxygen species, which increases the nuclear-isolated receptor Nur77 protein in Hep3B-HBX liver cancer cells expressing hepatitis B virus X protein (HBX). c-Jun amino-terminal kinase is activated to initiate apoptosis and promote hepatitis B virus clearance.

### 2.4. Anti-Tumor

Shikonin can be used to treat a diverse range of tumors, including lung, colon, breast, pancreatic, bladder, skin, and chondrosarcoma. Shikonin has numerous mechanisms of anti-tumor action, of which the ability of Shikonin to inhibit the cell division cycle and proliferation by inducing apoptosis, inducing the disruption of mitochondrial membrane potential, and anti-tumor angiogenesis has been focused on. Shikonin inhibits the viability, proliferation, invasion, and migration of non-small cell lung cancer A549 and PC9 cells, and induces apoptosis [37]. Shikonin can bind to the structural domain of the PAK1 kinase binding pocket, and Shikonin inhibits the activation of PAK1 and its downstream signaling pathway proteins to reduce the proliferation of pancreatic cancer cells and induce apoptosis, thus achieving a good therapeutic effect on pancreatic cancer [38]. The effect of Shikonin on pancreatic cancer is very favorable. Shikonin and its derivatives can inhibit phosphorylation-signal transducer and activator of transcription 3 (p-STAT3), and increase the activation of phosphorylation-protein kinase B (P-AKT), mitogen-activated protein kinases (MAPKs), phosphorylation-extracellular regulated protein kinases (P-ERK), phosphorylation-c-Jun N-terminal kinase (P-JNK), and P-p38 MAPK for chondrosarcoma treatment in a dose-dependent manner [39]. The anti-glioma mechanism of action of purslane is firstly by interfering with endoplasmic reticulum (ER) stress-mediated tumor apoptosis, and secondly by inducing mitochondrial outer membrane permeability (MOMP), triggering apoptosis in cancer cells [40]. Shikonin inhibits the proliferation of human melanoma cells by inducing apoptosis as mediated by the MAPK signaling pathway [41]. Treatment with Shikonin effectively inhibited the growth of human triple-negative breast cancer cell line MDA-MB-231 and mouse triple-negative breast cancer cell line 4T1 [42]. Shikonin inhibited cell progression and EMT and accelerated cell death by regulating the miR-106b/Recombinant Mothers Against Decapentaplegic Homolog 7 (SMAD7)/TGF-β signaling pathway to achieve the desired therapeutic effect in liver cancer treatment [43]. Ovarian cancer is the deadliest gynecological cancer in women. It was found that Shikonin inhibited the cell viability, migration, and invasive ability of type 2 ovarian cancer cells, and reduced the expression of CSC-related markers and the number of spherical colonies. It also reduced the tumorigenicity of Kuramochi cells in a xenograft model and induced anti-tumor effects [44]. Clinically, Boulos et al. [45] demonstrated using preliminary clinical trials that Shikonin has the potential to translate into clinical oncology. Clinical studies have found additive and synergistic interactions of Shikonin when used in combination with existing chemotherapeutic agents, immunotherapeutic approaches, radiation therapy, and other treatment modalities. Loading Shikonin with a number of nanomaterials can have a synergistic therapeutic effect on cancer. Nanoparticles have valuable pharmacokinetic properties, a large surface-to-mass ratio, high drug solubility, and a tunable drug-controlled release ability. Poly(lactic acid) (PLGA) biodegradable nanoparticles loaded with SK killed only epithelial ovarian cancer cells in the treatment of ovarian cancer but did not induce strong cytotoxicity in normal ovarian cells, endothelial MS1 cells, or lymphocytes. Therefore, these nanoparticles can be used as a new drug therapy against solid tumors [46]. The anti-tumor mechanisms of action of comedones are diverse, and with the in-depth study of their anti-tumor effects, it is believed that they can become a new and practical anti-tumor drug (Table 1).

### 2.5. Anti-Hepatic Injury

Liver injury is pathological damage to liver tissue caused by various biological, chemical, and physical factors. Repair of liver injury is the focus of liver research, and its mechanism involves many signaling pathways, cytokines, and transcription factors with a wide range of mechanisms of action. Acetaminophen (APAP) overdose can cause acute liver injury and lead to fatal liver injury. APAP, a modeling drug for liver injury, can lead to the production of inflammatory factors in the liver. In addition, pretreatment with Shikonin attenuates concanavalin A (CON-A)-induced acute liver injury by inhibiting the activation of the JNK pathway [47]. Pretreatment with Shikonin also inhibited the elevation of serum levels of alanine aminotransferase (ALT), aspartate aminotransferase (AST), and lactate dehydrogenase (LDH) in liver-injured mice, and significantly reduced APAP-induced histological changes in the liver tissue [48]. Purpurin treatment significantly reduced the production of serum TNF-α, IL-1β, IL-6, and IFN-γ inflammatory cytokines; decreased the serum levels of ALT, AST, MPO, and ROS in the liver, as well as their effects on histopathology; inhibited the phosphorylation of JNK1/2, ERK1/2, p38, and nuclear factor kappa B (NF-κB); and inhibited the phosphorylation and degradation of IκBα, thereby effectively treating the acute liver injury caused by intraperitoneal injection of LPS/D-GalN [49]. Shikonin also attenuated LPS/D-GalN-induced liver injury by inhibiting the TLR4 signaling pathway [50]. It was shown that Shikonin inhibited APAP liver injury by up-regulating Nrf2 via the PI3K/Akt/GSK-3β pathway. Therefore, Shikonin may be a promising candidate against APAP-induced liver injury [51].

Liver fibrosis is a process of tissue repair that occurs after various types of chronic liver injury and, if not effectively treated, can progress to cirrhosis, portal hypertension, and even liver cancer. Shikonin has anti-inflammatory, antiviral, and anti-tumor properties. In addition, Shikonin has anti-tissue-fibrosis and anti-organ-fibrosis effects. Shikonin promotes apoptosis of the LX-2 cells via the PAF–MAPK axis and inhibits autophagy, thus blocking the development of fibrosis [52]. Liu et al. [53] conducted a study using intraperitoneal injection of CCl_4_ and bile duct ligation to establish a liver fibrosis model in male C57 mice. The results showed that Shikonin can down-regulate the expression of transforming growth factor-β1, maintain the normal balance of metalloproteinase-2 and tissue inhibitor of metalloproteinase-1, and significantly inhibit the activation and extracellular matrix formation of hepatic stellate cells. These findings suggest that Shikonin has a potential inhibitory mechanism for both liver injury and liver fibrosis, making it a promising candidate for liver protection.

### 2.6. Promoting Wound Healing

*Lithospermum erythrorhizon* is a Chinese herb which has been used in China for over 2000 years. It is sweet, cool, and salty in flavor with the effects of cooling and activating the blood, clearing rashes, detoxifying, and treating sores. In addition, it is often used in the treatment of measles, spot sores, eczema, and burns. Shikonin has the pharmacological effect of promoting wound healing when used as a topical treatment in traditional Chinese medicine: its effect on skin repair is particularly prominent, and it is often made into a paste or infused oil. Shikonin/Shikonin dimers isolated from bark extracts had wound-healing-promoting effects on incisional wounds in albino rats [54]. During the second wound healing process in dogs, the side of the tissue treated with enantiomeric naphthoquinones alkannins and Shikonin (A/S) ointment had a significantly higher mean LDF value and higher collagen and epithelial thickness score compared to the effects of treatment with Ringer’s solution of lactic acid [55]. β-acetoxyisovaleryl alkannin (AAN-II), a purslane derivative, promotes ulcer healing by inhibiting the inflammatory response and promoting fibroblast proliferation and angiogenic factor secretion. Moreover, AAN-II can promote the healing of pressure-induced venous skin ulcers by activating TGF-β/drosophila mothers against decapentaplegic protein-3 (Smad3) signaling in fibroblasts [56]. Shikonin also promotes wound healing in Vibrio traumaticus-infected mice by promoting the formation of granulation tissue, hair follicles, and sebaceous glands, epithelial cell regeneration, and epidermal growth factor production [57]. A Shikonin analog promotes granulation tissue formation, including cell migration, angiogenesis, collagen production, and re-epithelialization. Shikonin increases the expression of basic fibroblast growth factor (bFGF), thereby promoting wound healing [58]. Shikonin promotes wound healing in gingival tissues by promoting recombinant hepatocyte growth factor (HGF) proliferation, migration, type I collagen and FN synthesis, and vascular endothelial growth factor (VEGF) and FN expression via the ERK 1/2 signaling pathway [59]. Shu et al. [60] developed a novel liposome containing Shikonin to improve the anti-methicillin-resistant *Staphylococcus aureus* effect and tested its beneficial wound-healing effect. This Shikonin liposome controls infection by inhibiting bacterial activity, modulates the inhibitor of the nuclear factor kappa B-α (IκB-α)/NF-κB signaling pathway to attenuate inflammatory infiltration, and promotes burn wound repair. Shikonin has always played an important therapeutic role in the skin, but more gaps in the mechanism of action of its skin repair need to be further explored.

### 2.7. Reduce Cognitive and Behavioral Disorders

Parkinson’s disease (PD) is the second most common neurodegenerative disease. Shikonin plays a protective role in age-related diseases. Guo et al. [61] investigated the biological functions of Shikonin and its mechanism of action in the pathogenesis of PD. Shikonin attenuated nigrostriatal dopaminergic neuron death and attenuated the neuroinflammation and nigrostriatal oxidative stress induced by the neurotoxin 1-methyl-4-phenyl-1,2,3,6-tetrahydropyridine (MPTP), which could ameliorate nigrostriatal neuronal damage and inhibit astrocyte activation. Shikonin inhibits oxidative stress and neuroinflammation by regulating the AKT/ERK/JNK/NF-κB pathway and improves apoptosis of the dopaminergic neurons in PD patients, which improves upon the evidence of Parkinson’s disease-induced pathology, as well as cognitive deficits.

## 3. Progress of Research Related to the Treatment of Skin Diseases Using Shikonin

### 3.1. Dermatitis

Shikonin has excellent anti-inflammatory properties. Atopic dermatitis (AD), commonly known as “eczema”, is easily mistaken by the general public for a minor dermatologic condition with a simple and unknown mechanism, thus neglecting treatment of the disease and its comorbidities, leading to exacerbation of symptoms, which seriously affects quality of life and imposes a heavy economic burden. Shikonin has good inhibitory or ameliorative effects on characteristic dermatitis. Oh et al. [62] found that red Shikonin (LE) could be a potential therapeutic agent for AD by regulating the Th1/Th2 immune balance and restoring the skin barrier’s function. Choi et al. [63] used low-temperature argon plasma (LTAP) as an adjunctive partner to topically applied yellow en cream (JO) to examine an AD mice model. The results revealed that LTAP–JO combination treatment blocked mediated NFκB/recombinant (RelA) activation and could significantly suppress the AD phenotype. Ku et al. [64] found that Jawoogo ointment (Shikonin being the main ingredient) reduced the skin’s thickness and improved the infiltration of inflammatory cells, mast cells, and CD4+ cells in dinitrochlorobenzene-induced atopic dermatitis-like BALB/C mice. The ointment reduced the mRNA levels of IL-2, IL-4, IL-13, and TNF-α in sensitized skin, and the mechanism of inhibition may be via the MAPK and NF-κB pathways. Yen et al. [65] found that Shikonin inhibited the expression of dp-2-induced cytokines (IL-6, IL-9, and IL-17A) and chemokines in the dendritic cells of patients with atopic dermatitis and that its inhibitory effect on the expression of IL-9, MIP-1β, and CCL5 was stronger than that of dexamethasone. Shikonin can promote the formation of granulomatous tissue and induce the neovascularization of granulomatous tissue in the skin tissue of rats. Yan et al. [66] found that Shikonin promoted the growth of human keratinocytes and concluded that Shikonin may promote skin wound healing by inhibiting skin inflammation via the inhibition of proteasomes. Shikonin also exerts anti-inflammatory effects by inhibiting the expression of the orphan nuclear receptor Nr4a family gene, a new proto-oncogenic calmodulin phosphatase inhibitor in mast cells [67]. The discovery of additional anti-inflammatory mechanisms will provide us with more ideas for the treatment of dermatitis using Shikonin, which will allow us to maximize the efficacy of Shikonin.

### 3.2. Psoriasis

**Figure 4:**
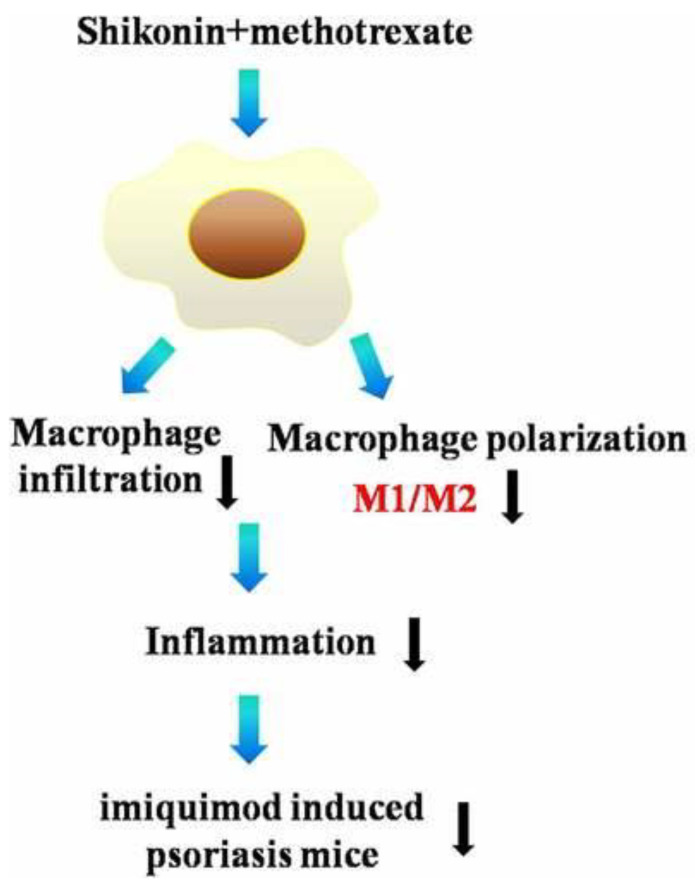
Alizarin combined with methotrexate modulates M1/M2 polarization [73].

Psoriasis is an immune-mediated inflammatory disease that develops under the combined effects of genetics and environmental factors, and is clinically characterized by scaly erythematous plaques; this condition is mostly mild to moderate. It is characterized by a long course and easy recurrence, and predominantly affects the skin and joints. The therapeutic effects of Shikonin or Shikonin-based combinations on psoriasis have been most widely studied [68]. The most widely studied therapeutic effect of Shikonin or Shikonin-based compound preparations on psoriasis is that Shikonin can effectively inhibit the activation of JAK/STAT3 and up-regulate the expression of recombinant CCAAT/enhancer-binding protein delta (CEBPD), thus inhibiting the proliferation and apoptosis of psoriatic keratinocytes [69]. Zhang et al. [70] found that Shikonin could effectively promote Treg cell differentiation in vitro by inhibiting the AKT/mTOR pathway, while Shikonin significantly increased the expression of Foxp3 in the skin of psoriatic mice. IL-17 is involved in the pathogenesis of psoriasis and promotes the proliferation of epidermal keratinocytes via signal transducer and activation of transcription activator 3 (STAT3). Shikonin inhibits IL-17-induced vascular endothelial growth factor expression by regulating the JAK/STAT3 signaling pathway. Shikonin reversed IL-17-mediated downregulation of the tumor suppressor CEBPD in HaCaT cells [71]. The main active components of Shikonin, Shikonin (Shikonin) and β, β-dimethylacryloylalkylin (DMA), have strong anti-inflammatory effects. Wang et al. [72] found that an herbal formula based on red Shikonin significantly improved psoriasis dermatitis and significantly reduced psoriasis area and severity index (PASI) scores when treating patients with psoriasis. The decrease in epidermal thickness after Shikonin or DMA treatment was greater than that of the control group. In praziquimod-induced psoriasis mice, vincristine combined with methotrexate exerted a protective effect by reducing erythema and the PASI scores, decreasing the backer scores and epidermal thickness, and especially modulating macrophage polarization. In LPS-stimulated RAW264.7 cells, Shikonin combined with methotrexate modulated M1/M2 polarization and altered the levels of M1 markers (Figure 4) [73]. Exploiting the therapeutic role of Shikonin may help more patients reduce the pain and psychological burden caused by psoriasis.

### 3.3. Skin Cancer

Hepatocellular carcinoma is a malignant tumor of the skin, which includes squamous cell carcinoma, basal cell carcinoma, malignant melanoma, malignant lymphoma, idiopathic hemorrhagic sarcoma, sweat adenocarcinoma, and angiosarcoma. Skin cancers include primary carcinomas and those that metastasize to the skin from other parts of the body, secondary primary skin cancers, and secondary skin cancers that metastasize to the skin from other parts of the body. Primary skin cancers commonly include basal cell carcinoma, squamous carcinoma in situ (Bowen’s disease), squamous cell carcinoma, eczema-like carcinoma, and malignant melanoma. Despite extensive research over centuries, the treatment of malignant melanoma remains challenging because of its mostly insignificant metastatic spread and rapid growth rate. Therefore, the discovery of new drug precursors is an important goal. To test whether PKM2 might be a target for prevention, Li et al. [74] conducted a study of chemically induced skin cancer in mice using Shikonin, a natural product of Shikonin root, and a specific inhibitor of human pyruvate kinase (PKM2). The results showed that Shikonin treatment inhibited skin tumor formation. Shikonin inhibited cell proliferation but did not induce apoptosis, and instead of inhibiting PKM2, Shikonin inhibited the activating transcription factor 2 (ATF2) pathway in skin carcinogenesis. Kretschmer et al. [75] prepared 31 mainly novel Shikonin derivatives and screened them in different melanoma cell lines (WM9, WM164 and MUG-Mel2 cells) using the XTT activity assay to identify a novel derivative of Shikonin with higher activity. The compound induced apoptosis and reduced apoptosis in the G1 phase cells and exhibited cytotoxicity against non-tumorigenic cells. STAT3 signaling promotes melanoma genesis and progression and is a potent target for melanoma therapy. Cao et al. [76] assessed the anti-melanoma activity of Shikonin and explored the role of the STAT3 signaling pathway. Shikonin was found to inhibit melanoma growth in cultured cells and a zebrafish xenograft model. So, Shikonin reduces the nuclear localization of STAT3 by inhibiting its phosphorylation and homodimerization. Cui et al. [77] found that the Shikonin derivative DMAKO-20 could exhibit selective cytotoxic effects on melanoma cells via recombinant cytochrome P450 1B1 (CYP1B1)-mediated activation. Using DMAKO-20 as a lead compound, further structural optimization may provide new drug entities for the treatment of malignant skin cancer. In summary, Shikonin is a potential drug for the treatment of skin cancer.

### 3.4. Scarring

Proliferative scar formation is a very common clinical condition that results from a decrease in the number of apoptotic fibroblasts and excessive collagen production during scar formation after wound healing. Shikonin has several mechanisms of action for the treatment of scarring. Shikonin preferentially inhibits cell proliferation and induces apoptosis in fibroblasts without affecting keratinocyte function. In addition, Shikonin’s apoptosis-inducing ability may be triggered via the MAPK and B-cell lymphoma-2 (Bcl-2)/caspase-3 signaling pathways [78]. Shikonin could inhibit fibrosis and migration of hypertrophic scar fibroblasts by down-regulating the expression of microRNA-382-5p [79]. Shikonin has apoptosis-inducing effects on human proliferative scar tissue fibroblasts [80]; Shikonin inhibited the expression of p63, cytokeratin 10, α-smooth muscle actin, transforming growth factor β1, and type I collagen during proliferative scar formation [81].

### 3.5. New Dosage Forms of Shikonin in Diseases

Due to the low water solubility of Shikonin, its bioavailability is limited, which hinders its application. In recent years, researchers have extensively studied various emerging dosage forms to enhance the bioavailability of poorly soluble drugs [82,83,84]. Liposomes, which are nanoparticles with a bilayer structure similar to phospholipids, can encapsulate drugs within their interior to improve drugs’ bioavailability [85]. Wen et al. [86] prepared Shikonin liposomes using a film dispersion method and modified them with RGD (RGD-SSLs-SHK) to overcome the issue of poor water solubility. The experimental results demonstrate that RGD-SSLs-SHK exhibits excellent physical and chemical properties in terms of particle size, zeta potential, encapsulation efficiency, and delayed release time. Furthermore, it significantly inhibits the proliferation of breast cancer cells, indicating that loading Shikonin via liposomes holds promise as a nanoformulation for disease treatment. Other studies have used three surfactants (saponin, sophorolipid, and rhamnolipid) to modify Shikonin nanoparticles. The results show that saponin- and sophorolipid-coated Shikonin nanoparticles have much higher encapsulation efficiencies (97.6% and 97.3%, respectively) compared to rhamnolipid-coated Shikonin nanoparticles (19.0%). Additionally, saponin- and sophorolipid-coated Shikoninn nanoparticles improve its in vitro bioavailability [87]. Micelles, which are self-assembled core–shell nanocarriers made of lipids or polymers, offer advantages such as ease of production, high drug loading capacity (up to 30%), small particle size (less than 200 nm), and modifiability. Consequently, the use of micelles in oral formulations is gaining popularity [88]. Li et al. [89] investigated the effect of methoxy polyethylene glycol-β-gathering (MPEG-PCL) micelles loaded with Shikonin on inflammatory cytokine-induced endothelial mesenchymal transition (EndMT). The experimental results demonstrated that MPEG-PCL micelles significantly enhanced the cellular absorption of Shikonin. Real-time PCR analysis revealed that co-treatment with TNF-α and IL-1β successfully induced EndMT in HUVECs, while Shikonin and Shikonin-loaded MPEG-PCL micelles effectively inhibited this process.

In conclusion, enhancing the bioavailability of Shikonin using nanotechnology and expanding its application is a viable strategy. Future research can focus on further enhancing the bioavailability of Shikonin using nanotechnology while also exploring its biological activity. However, it is crucial to investigate the biocompatibility of nanoparticles during the application process, as this is one of the key factors to consider.

## 4. Conclusions and Outlook

In summary, Shikonin has a wide range of pharmacological activities, such as anti-inflammatory, antibacterial, antiviral, and anti-tumor properties, and promotes skin healing. Among the many pharmacological activities of Shikonin, its anti-inflammatory and anti-tumor activities are outstanding, which are the hotspots and focuses of current research, and are expected to be applied to new drugs for treating major diseases in the future. The discovery of other pharmacological effects of Shikonin is the focus of future research. The wide range of pharmacological activities of Shikonin is important for the treatment of skin diseases, and Shikonin has an important role in the treatment of dermatitis, psoriasis, skin cancer, scarring, and so on. However, the dosage form of the traditional Chinese medicine Shikonin developed as a new drug is relatively single, mostly developed as cream and oil. It is hoped that more new formulations of Shikonin can be developed in the future. Moreover, the anti-inflammatory/antioxidant properties, barrier repair, regulation of cell proliferation and apoptosis, etc. of Shikonin itself, and the transdermal reaction and synergistic effect of traditional Chinese medicine compounds’ topical preparation, their prevention of pigmentation, and their antibacterial effects, among other things, in the development of certain skin diseases, all mean that Shikonin will be used on the skin to play a greater effect in skin restoration. It is believed that with the progress of science and technology and the continuous supplementation and improvement of the theory of traditional Chinese medicine, the role of Shikonin in skin repair can be better utilized and amplified to help the human skin restore health. Thus, the development of more research on the pharmacological effects of Shikonin and dosage forms is essential.

## Figures and Tables

**Table 1:** Mechanisms of anti-tumor effect of Shikonin.

Key Numbers	Tumor Type	Mechanism of Action	References
1	Lung cancer	Inhibits the viability, proliferation, invasion, and migration of non-small cell lung cancer A549 and PC9 cells.	[37]
2	Pancreatic cancer	Shikonin inhibits the activation of PAK1 and its downstream signaling pathway proteins.	[38]
3	Chondrosarcoma	It can inhibit pSTAT3 and increase pAKT, MAPKs, pERK, pJNK, and p-p38 MAPK.	[39]
4	Glioma	Tumor apoptosis mediated by interference with endoplasmic reticulum (ER) stress; induction of mitochondrial outer membrane permeability (MOMP) triggers apoptosis of cancer cells.	[40]
5	Melanoma	Apoptosis was induced by the MAPK pathway.	[41]
6	Triple-negative breast cancer	The growth of human triple-negative breast cancer cell line MDA-MB-231 and mouse triple-negative breast cancer cell line 4T1 was inhibited.	[42]
7	Liver cancer	The miR-106b/SMAD7/TGF-β signaling pathway inhibits cell progression and EMT and accelerates cell death.	[43]
8	Ovarian cancer	Shikonin inhibited cell viability, migration, and invasion of type 2 ovarian cancer cells. The expression of CSC-related markers and the number of cocci colonies were reduced. The tumorigenicity of Kuramochi cells was also reduced.	[44]

## Data Availability

The data supporting the findings are available from the corresponding authors upon reasonable request.
